# Temperament and character profiles of patients with chronic migraine and medication-overuse headache

**DOI:** 10.1186/1129-2377-14-S1-P178

**Published:** 2013-02-21

**Authors:** Y Watanabe, H Tateno, K Hirata

**Affiliations:** 1 Dokkyo Medical University, Japan

## Background and purpose

The frequency of psychiatric co-morbidity or psychological distress has been reported to be high among patients with chronic migraine (CM) and medication-overuse headache (MOH). However, the personality profile of such patients is unclear. The objective of this study was to assess the personality profile of a sample of Japanese patients with migraine by using the Temperament and Character Inventory (TCI).

## Methods

This was a cross-sectional study that included 138 adult patients with migraine. In total, 100 age-, sex-, and educational level-matched healthy subjects were selected as a control group. The patients were divided into 3 groups according to the second edition of the International Headache Classification: those with episodic migraine (EM; n = 79), those with CM (n = 28), and those with MOH (n = 31). The patients in the 3 migraine groups and individuals in the control group completed psychometric questionnaires, including the TCI and Beck Depression Inventory (BDI).

## Results

The mean BDI score and the dimension harm avoidance (HA) score of the patients in the 3 migraine groups were significantly higher than those of individuals in the control group. The mean BDI and HA scores were the highest in the MOH group among the 3 migraine groups.

## Conclusion

Our results indicate serotonergic involvement in the physiopathology of migraine. In addition, it might risk factor of the migraine chronicity that BDI and HA score are high.

## Competing interests

The authors have no potential conflicts of interest to disclose.
